# Gesture Recognition in Robotic Surgery with Multimodal Attention

**DOI:** 10.1109/TMI.2022.3147640

**Published:** 2022-06-30

**Authors:** Beatrice van Amsterdam, Isabel Funke, Eddie Edwards, Stefanie Speidel, Justin Collins, Ashwin Sridhar, John Kelly, Matthew J. Clarkson, Danail Stoyanov

**Affiliations:** Wellcome/EPSRC Centre for Interventional and Surgical Sciences (WEISS), University College London, UK; Division of Translational Surgical Oncology, National Center for Tumor Diseases (NCT), Partner Site Dresden, Dresden, Germany, and with the Centre for Tactile Internet with Human-in-the-Loop (CeTI), TU Dresden, Dresden, Germany; Wellcome/EPSRC Centre for Interventional and Surgical Sciences (WEISS), University College London, UK; Division of Translational Surgical Oncology, National Center for Tumor Diseases (NCT), Partner Site Dresden, Dresden, Germany, and with the Centre for Tactile Internet with Human-in-the-Loop (CeTI), TU Dresden, Dresden, Germany; Department of Urooncology, University College London Hospital NHS Foundation Trust, London, UK; Department of Urooncology, University College London Hospital NHS Foundation Trust, London, UK; Department of Urooncology, University College London Hospital NHS Foundation Trust, London, UK; Wellcome/EPSRC Centre for Interventional and Surgical Sciences (WEISS), University College London, UK; Wellcome/EPSRC Centre for Interventional and Surgical Sciences (WEISS), University College London, UK

**Keywords:** surgical gesture recognition, multimodal attention, surgical data science, robotic surgery

## Abstract

Automatically recognising surgical gestures from surgical data is an important building block of automated activity recognition and analytics, technical skill assessment, intra-operative assistance and eventually robotic automation. The complexity of articulated instrument trajectories and the inherent variability due to surgical style and patient anatomy make analysis and fine-grained segmentation of surgical motion patterns from robot kinematics alone very difficult. Surgical video provides crucial information from the surgical site with context for the kinematic data and the interaction between the instruments and tissue. Yet sensor fusion between the robot data and surgical video stream is non-trivial because the data have different frequency, dimensions and discriminative capability. In this paper, we integrate multimodal attention mechanisms in a two-stream temporal convolutional network to compute relevance scores and weight kinematic and visual feature representations dynamically in time, aiming to aid multimodal network training and achieve effective sensor fusion. We report the results of our system on the JIGSAWS benchmark dataset and on a new in vivo dataset of suturing segments from robotic prostatectomy procedures. Our results are promising and obtain multimodal prediction sequences with higher accuracy and better temporal structure than corresponding unimodal solutions. Visualization of attention scores also gives physically interpretable insights on network understanding of strengths and weaknesses of each sensor.

## I Introduction

SURGICAL robots are now an established part of clinical practice, particularly in minimally invasive surgery, where surgeons especially benefit from the enhanced instrumentation, visualisation and ergonomics during the procedure [1]. In addition to advantages for the patient and clinical team, the surgical robot system is a complex platform and potentially captures large amounts of unique data from the surgical procedure that can be used to develop artificial intelligence solutions for the future surgical operating room benefiting from computer assisted interventions (CAI) [2]. Robotic systems in fact capture digital videos as well as instrument kinematic trajectories, instrument types and other system signals during surgical interventions, enabling more in-depth analysis of surgical motion and activity than with traditional instrumentation or video alone.

**Fig. 1:**
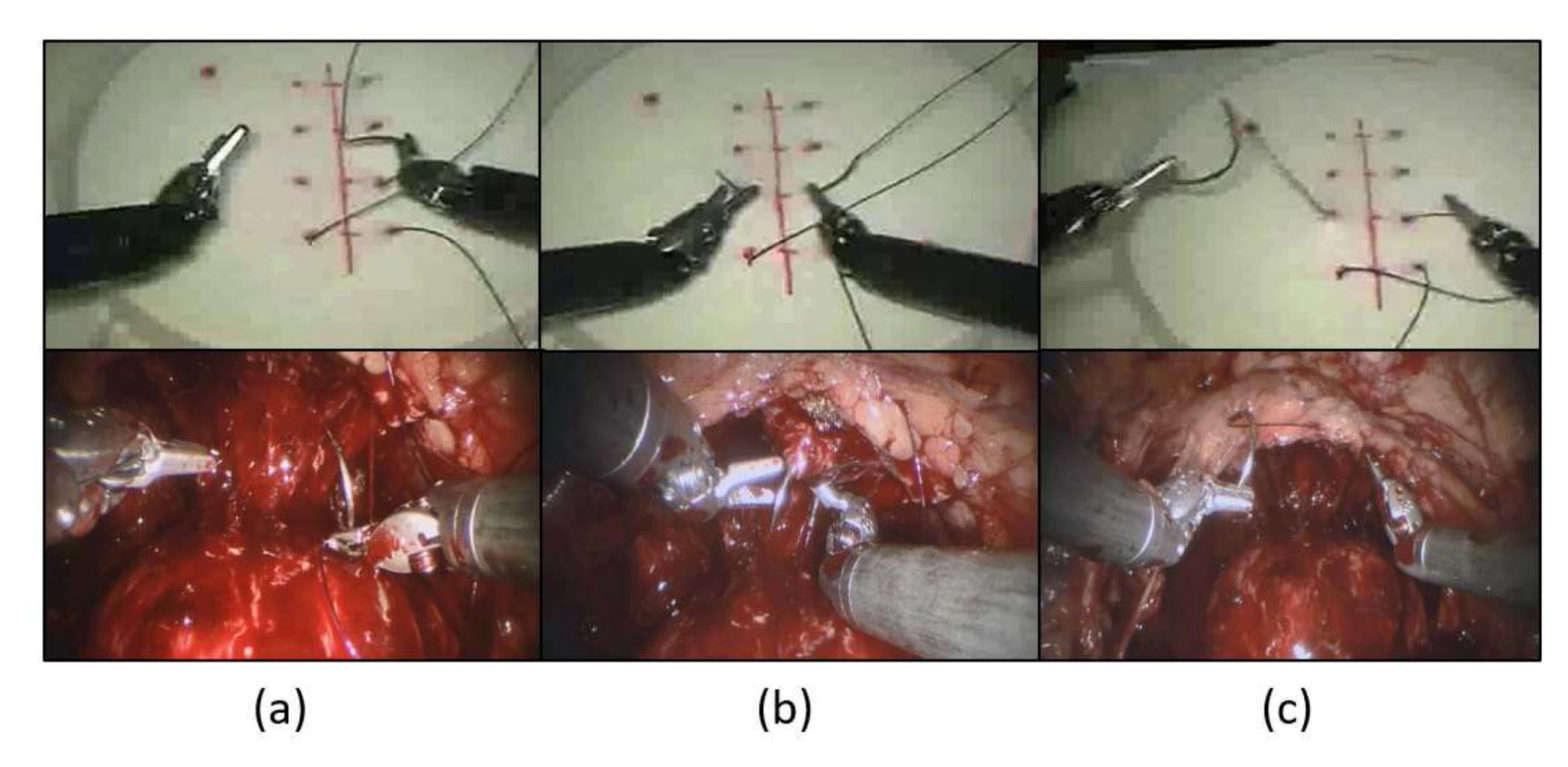
Surgeme examples in phantom and real surgical environments: (a) *positioning needle tip on insertion point*, (b) *pushing needle through the tissue*, (c) *pulling needle out of tissue*. Snapshots on top from JIGSAWS [3].

Surgical motion, activity and process understanding are fundamental concepts in surgical data science (SDS) and CAI, representing the cornerstone of various implementations of pre-, intra- and post-operative clinical support systems [4]. Analysis of surgical motion and robot kinematics is often based on decomposition into pre-defined action units, called “surgical gestures” or “surgemes” (Fig. 1), representing finegrained motion segments performed with a specific surgical purpose (e.g. grabbing the needle, pushing the needle through the tissue). Automatic segmentation of surgical demonstrations into fine-grained gestures finds application in technical skill assessment and development [3], [5], [6], as it allows a system to provide surgical trainees with quantitative and gesture-specific feedback, as well as surgical automation, where modular blocks of motion can be learnt, composed and reused more easily than long surgical tasks [7]. If performed in real-time, gesture recognition can also be exploited for any application based on context-awareness, such as workflow monitoring, error detection and intra-operative assistance [8], [9]. Linking surgical actions to patient outcomes can finally give new insights for strategy optimization [10].

Fine-grained analysis of surgical motion however presents significant challenges, due to the complexity of surgical trajectories and the presence of multiple independent variability sources, such as user-specific surgical style and skill level. The same gesture can also be used across different surgical phases and procedures, where contextual features such as instrument type and anatomical site are generally different but can hardly be exploited to discriminate between fine motions. The combination of these variability factors leads to alterations in the kinematic, temporal and sequential properties of surgical actions in a surgeon-, patient- and task-specific manner [11].

A promising but relatively unexplored strategy to enhance available recognition systems and improve their performance is represented by the integration of synchronous data streams recorded from the robotic platform, which often encode complementary information. Kinematic data, for example, represent the robotic system configuration and its motion in space, while endoscopic videos contain information about the environment, other tools and objects (e.g. needle, assistant’s tools) and their interaction in the surgical scene. Robust sensor fusion is however non-trivial because each sensor is subject to specific noise sources and has different predictive power in different contexts [12]. As an illustration, kinematic information is expected to return more accurate predictions when the view on the surgical scene is occluded or the surgical instruments move out of the camera’s field-of-view. On the other side, visual features are essential to discriminate gestures with similar motion pattern performed on different anatomical structures or with different surgical tools and objects. Balancing uni-modal information in a timely manner, that is with stronger focus on the most reliable modality at each time stamp, could thus be key to rectify unimodal prediction errors and obtain robust gesture recognition from multiple data sources [12], [13]. While previous work mostly relied on unimodal data or plain concatenation of uni-modal features, analysis of more complex interactions between visual and kinematic streams has been rarely investigated [14], [15], especially in real-case scenarios where robot kinematic information is not always freely accessible.

In this paper, we explore using attention mechanisms, which have gained much popularity in text data processing [16], to compute relevance scores and weight high-level kinematic and visual feature representations dynamically in time, aiming to aid multimodal network training and achieve effective sensor fusion between video and kinematics. The proposed attention modules are embedded in a two-stream temporal convolutional network, but can in principle be used with a variety of two-stream recognition systems.

We evaluate our proposed system on the JIGSAWS benchmark dataset [3], [11] and on a new dataset of suturing demonstrations from in vivo robotic prostatectomy interventions and we provide promising comparisons to the state-of-the-art. In our new clinical data, fine-grained analysis and multimodal fusion are particularly challenging due to the complexity of the surgical environment and larger number of noise sources and variability factors. This represents an important first step towards translation of current research in gesture recognition systems and model deployment in real surgical scenarios, which has been hindered thus far by the lack of large and realistic open-source datasets essential for deep learning solutions.

In summary, our contributions include:

- Integrating multimodal attention mechanisms in a surgical gesture recognition system with the aim of weighting kinematic and visual feature representations dynamically in time, thus aiding multimodal network training and achieving effective sensor fusion.
- Introducing a new in vivo dataset for surgical gesture recognition made of suturing segments from robotic prostatectomy procedures. The video dataset and annotations will be made available for research purposes at https://www.ucl.ac.uk/interventional-surgical-sciences/weiss-open-data-server.
- Experimentally showing the effectiveness of attentionbased multimodal fusion on the JIGSAWS benchmark dataset as well as on our challenging in vivo data.

## II Related Work

### A Surgical Gesture Recognition - Temporal Models

Research on automatic recognition of surgical gestures has often drawn inspiration from state-of-the-art models for speech recognition and machine translation, as surgical demonstrations obey task-specific, probabilistic action grammars in a similar way as syntactic rules regulate the natural language flow. Probabilistic graphical models such as hidden Markov models [17] and conditional random fields [18], [19] have been extensively used in early research stages to learn such probabilistic grammar from video and kinematic data.

Current research is focused on more powerful solutions based on deep learning and in particular on temporal convolutional and recurrent models, which work efficiently on low-dimensional input data such as kinematic trajectories or high-level visual features encoded with 2D [19] or 3D convolutional neural networks (CNNs) [20].

Temporal convolutions are often used in encoder-decoder networks where action predictions are generated simultaneously at all time stamps. Temporal Convolution Network (TCN) [21], [22] uses a cascade of temporal convolutions and pooling/upsampling layers to capture temporal correlations in the input data at multiple hierarchical levels. To avoid loss of fine-grained information, two-stream solutions process the data at two different temporal scales and merge information at multiple processing levels [23], [24]. The same issue can be tackled by stacking multiple layers of atrous temporal convolutions with increasing dilation factor, thus increasing the network temporal receptive field without pooling operations [25], [26].

Recurrent models such as Long Short-Term Memory (LSTMs) [27] and Multi-Scale Recurrent Neural Network (MS-RNN) [28], on the other side, are built around memory cells able to store long-term information of past observations. Hybrid models based on temporal convolutions and recurrent structures, either combined sequentially [29] or in parallel [14], have shown good recognition capabilities.

### B Surgical Gesture Recognition - Sensor Fusion

Joint learning and fusion of multimodal data (video, kinematics and optical flow) for gesture recognition has been investigated in the literature [30], [31], giving insights that suggest the improved performance of multimodal models over their unimodal counterparts. Most related work approached sensor fusion through plain concatenation of uni-modal features [32]–[35], which could however be suboptimal due to differences in semantic and stochastic properties. Only a few studies have investigated more complex interactions between different data streams. Fusion-KV [14] consists of parallel recognition models operating on different data sources. Information fusion is performed at testing time, where individual predictions are weighted according to a voting scheme based on class-specific, uni-modal training performance. Class-specific weighting is however unsuitable to capture more detailed interactions and their evolution in time. Dynamic integration of high-level visual and kinematic embeddings has been achieved through a relational graph learning module (MRG-Net) [15], aimed at capturing joint knowledge to produce refined uni-modal embeddings. In a similar fashion, we use multimodal attention to seek timely multimodal cooperation and refine hidden representations for more accurate gesture recognition.

### C Attention-based Temporal Multimodal Learning

Temporal Multimodal Learning (TML) [13] aims at simultaneously fusing multimodal information and modelling temporal dynamics in sequential data. Attention-based approaches for TML have been explored for video classification [13], [36] and video captioning [12], [37] from visual, motion and audio signals.

A simple but efficient solution consists in obtaining a global representation of each input sequence through independent temporal models (e.g. LSTM) with attention, and then fuse these high-level representations for video classification [36]. The advantage is that each modality independently learns to pay attention to different temporal segments, while multimodal interactions are still captured in late processing stages.

More advanced fusion strategies extend the attention mechanism by not only localizing relevant temporal windows, but also weighting the contribution of each data modality dynamically in time [12], [13], [37]. Dynamic weights can be assigned to each modality based on the agreement between the current input and the previous multimodal representation for video classification [13], or with all the previously generated words for video captioning [12], [37]. In a similar fashion, our model dynamically adjusts the relative contribution of each input stream to generate better multimodal representations.

## III Datasets

### A JIGSAWS Dataset

The JHU-ISI Gesture and Skill Assessment Working Set (JIGSAWS) [3], [11] represents the benchmark dataset for surgical gesture recognition. It contains synchronized kinematic and video recordings of elementary surgical tasks (suturing, knot-tying, needle passing) executed by eight different surgeons using the da Vinci Surgical System (dVSS) [38]. With focus on the suturing task, all 39 available demonstrations have been manually segmented into fine-grained gestures according to a pre-defined dictionary of 10 action classes. Amendments to the original labels, rectifying 12 annotation errors, are reported in [39].

While JIGSAWS has been widely used by the research community for model development and comparison, it also shows some limitations which prevent the applicability of such methods to real-world surgeries. One is the limited size of the dataset (each demonstration only lasts around 1.5 minutes and contains approximately 20 gesture instances), which hinders robust training and testing of deep-learning-based recognition systems. Despite involving different users, lack of data diversity is apparent compared to real surgeries due to the standardised training environment and predetermined workflow structure, leading to similar instrument positions and directions of motion. Another problem is the lack of endoscopic motion and zoom, with all demonstrations observed from the same point of view. This leads to poor generalization to new endoscopic views in real-case scenarios, where camera motions are generally frequent. For technological advancement and translational research, future work requires testing on more realistic demonstrations with complex anatomies, camera motions, different illumination conditions, blood, specularities, occlusions and higher variability in action ordering and execution strategy.

### B RARP-45 Dataset

**Fig. 2:**
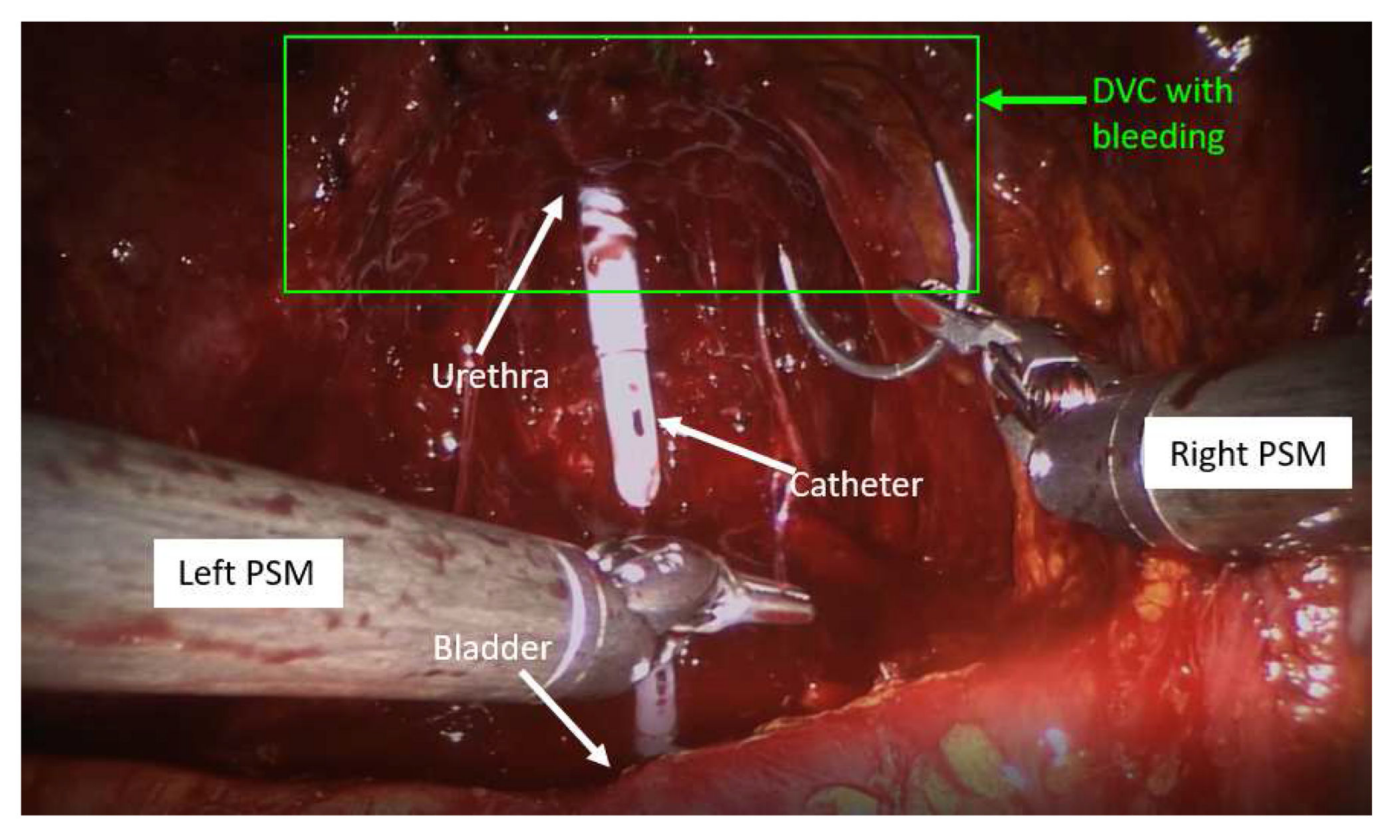
The dorsal vascular complex (DVC), an array of veins and arteries that carry blood to the penis, is sutured to keep bleeding under control during radical prostatectomy procedures.

In order to explore multimodal data integration in more challenging and realistic conditions, we collected a dataset of Robot-Assisted Radical Prostatectomies (RARP) performed by eight surgeons with different surgical seniority (experienced consultant, senior registrar and junior registrar) using a da Vinci Si Surgical System (Intuitive Surgical, Inc.) at the Westmoreland Street Hospital, London, UK, part of the University College London Hospitals NHS Trust^1^. Robotic radical prostatectomy is the surgery to remove the whole prostate gland and represents common treatment against clinically localised prostate cancer [40]. Its surgical workflow involves a first dissection phase, where the connection of the prostate to bladder and urethra are cut and the prostate is removed; the dorsal vascular complex (DVC), an array of veins and arteries that carry blood to the penis, is then sutured to keep bleeding under control (Fig. 2); finally the bladder and urethra are stitched back together. We carried out fine-grained analysis on the DVC suturing phase, which is more structured than preceding dissection phases and much less complex than the following anastomosis segment.

**Fig. 3:**
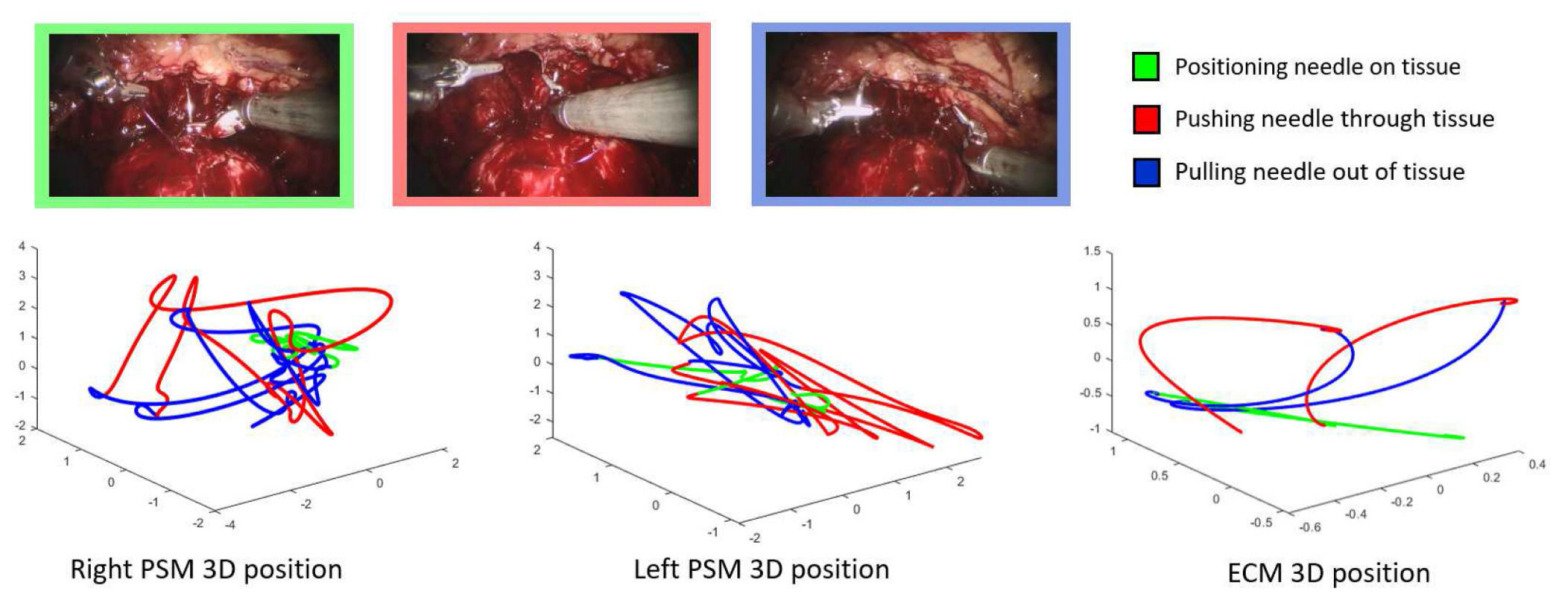
The RARP-45 dataset consists of synchronized video and kinematic data recorded from the da Vinci Si Surgical System during robotic radical prostatectomy surgery. Manual segmentation into fine-grained bi-manual actions was carried out on the DVC suturing phase of the procedure.

The data consist of synchronized video and kinematics (Fig. 3) captured from the robotic platform at 60 Hz and 50 Hz respectively using the dVLogger (Intuitive Surgical, Inc.). Kinematic features include pose and joint angles of three Patient Side Manipulators (PSMs), two Master Tool Manipulators (MTMs) and the Endoscopic Camera Manipulator (ECM). Videos were recorded from the endoscopic camera held by the ECM and used in the labelling process as well as for recognition. A dictionary of 7 fine-grained bi-manual gestures and a background class (Table I) was designed in collaboration with expert surgeons to guide manual segmentation of DVC suturing demonstrations from 45 different interventions. Annotations were created by a trained engineer as there was no discrepancy between clinical and non-clinical understanding of the surgical gestures employed in this study.

**Fig. 4:**
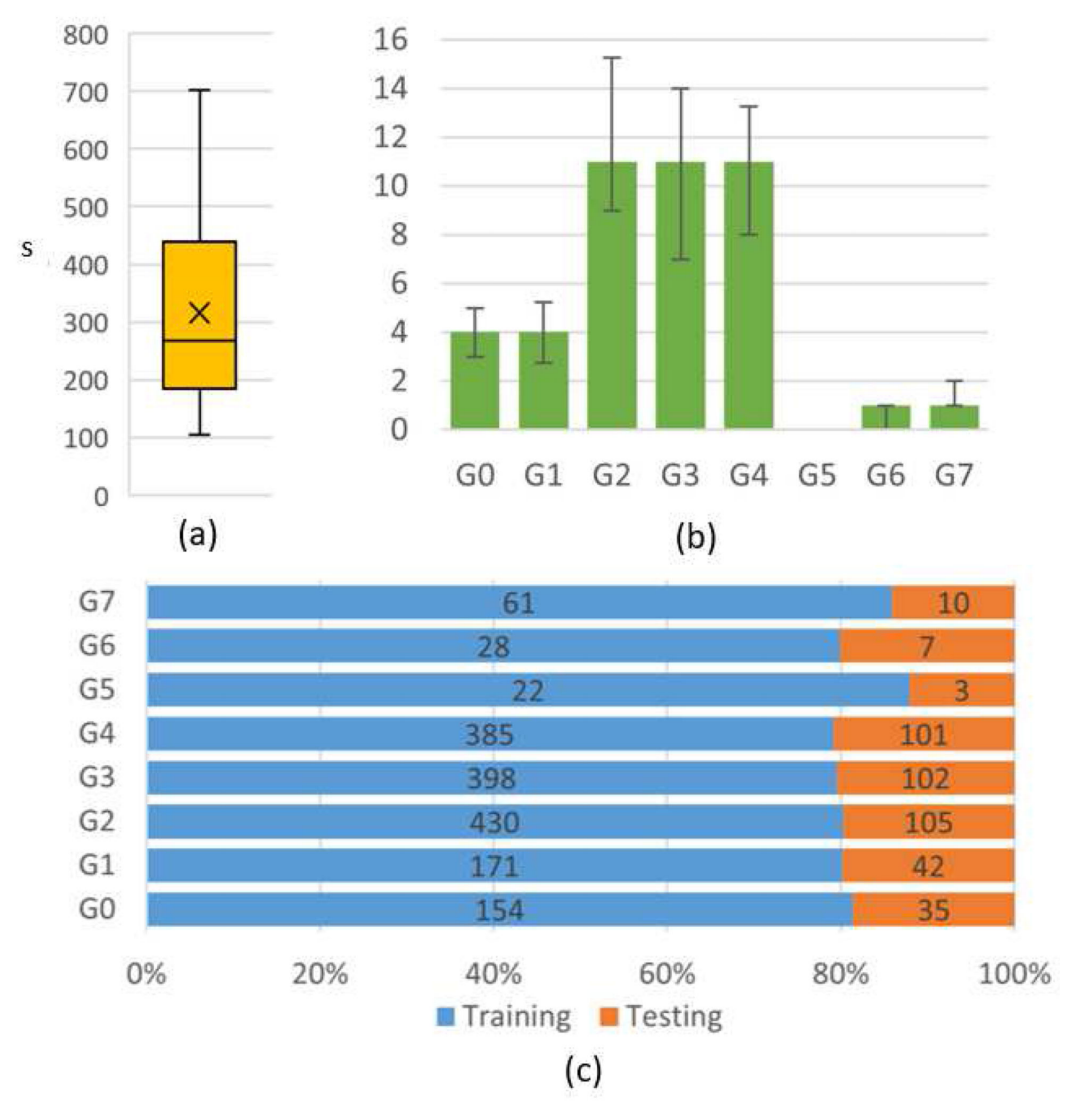
RARP-45 statistics. (a) Task duration variability (reported in seconds). Average duration is about 5 minutes, with large variability ranging from about 2 to 12 minutes. (b) Class distribution per sequence. Each bin represents the median class frequency over interventions, and error bars mark the 25th and 75th quantiles. Class G5 is absent in more than 75% of the interventions. (c) Train and test class distribution. Absolute frequencies are reported on the bins. Relative frequencies are homogeneous across all classes.

**Fig. 5:**
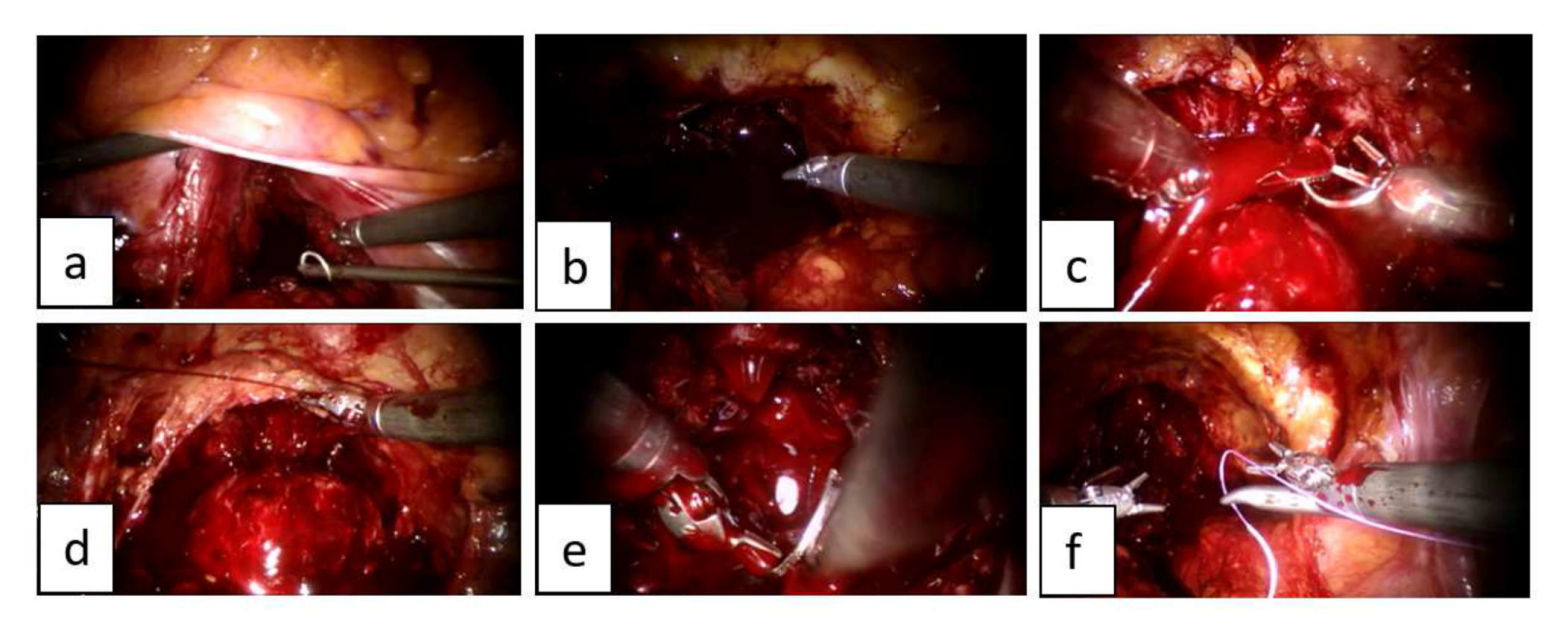
Examples of variability factors and noise sources which hinder robust gesture recognition in real-case scenarios: (a) environment variability due to patient-specific anatomical structure, (b) changes of illumination, (c) presence of blood, (d) tools out of view, (e) occlusions and self-occlusions, (f) interactions with surgical assistant.

Different trials show considerable variability in terms of total duration (Fig. 4a), as well as action count (Fig. 4b), ordering and kinematic properties. Such diversity is only partially operator-dependent, reflecting different surgical style and robotic surgical experience, but it is also linked to real-case variability factors such as patient-specific anatomical structure and tissue response (e.g. unexpected or excessive bleeding, which could prompt multiple gesture attempts or alter the surgical strategy). Other variability factors and noise sources which hinder robust gesture recognition in real-case scenarios include the presence of camera motions, changes of illumination, specularities, smoke and blood (Fig. 5). Given the endoscope proximity to the suturing site, surgical tools often fall out of the camera field-of-view or their line-of-sight is interrupted by obstructions or self obstructions, especially during interactions with the surgical assistant, which brings additional tools to the surgical scene and often leads to altered kinematic trajectories.

## IV Methods

### A Unimodal streams

**Fig. 6:**
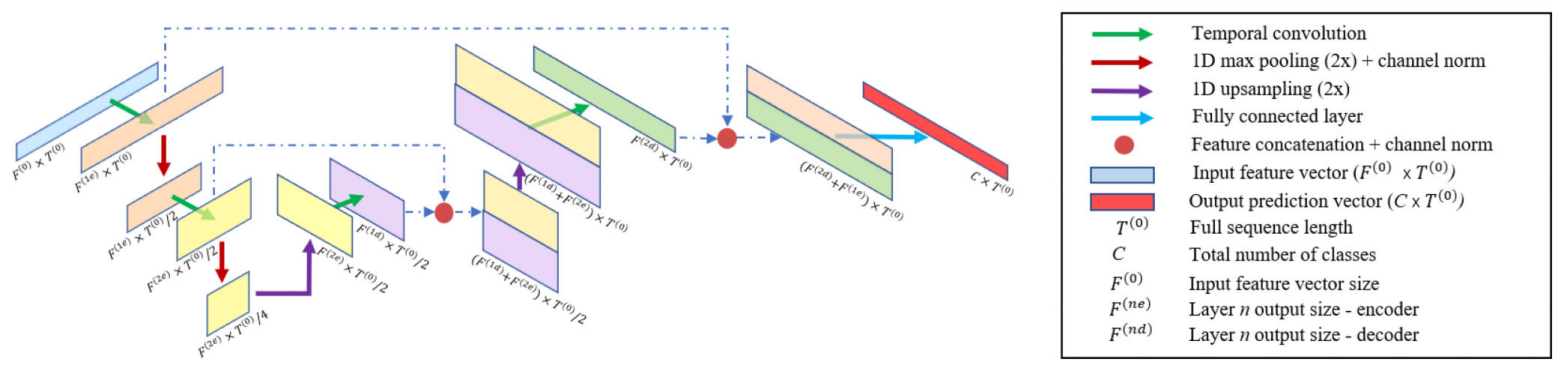
Unimodal baseline. A cascade of temporal convolutions and pooling layers to encode the input sequence at increasing levels of abstraction is followed by temporal convolutions and upsampling layers to gradually bring the data back to their original temporal resolution for frame-wise classification. Shortcut connections are introduced between encoding and decoding stages, where features from corresponding layers are concatenated to allow the propagation of low level contextual information to the high level layers. After each max pooling and feature concatenation layer, channel-wise feature normalization as well as temporal dropout are employed for regularization.

Our multimodal system is based on two parallel unimodal streams operating on high-level feature sequences derived from the original video and kinematic data (see Section IV-C). Each stream is a temporal convolutional network (TCN), composed of a contracting part (encoder) and an expansive part (decoder) (Fig. 6). The encoder consists of a cascade of temporal convolutions and pooling layers operating directly on the input time series, aimed at modelling kinematic or visual information at larger temporal scales and increasing levels of abstraction. Starting with input sequence *X*^(0)^ ∈ ℝ^*F*(0) × *T*(0)^, where *F*^(0)^ is the input feature dimension and *T*^(0)^ is the total sequence length, the intermediate feature sequence *X*^(l)^ ∈ ℝ^*F(l)*×*T(l)*^ at layer *l* ∈ [1, *L*] is computed at each time step *t* as:

(1) $${\hat{X}}_{t}^{\left(l\right)}=f\left(W^{\left(l\right)}*X_{t-\frac{c}{2}-1:t+\frac{c}{2}}^{\left(l-1\right)}+b^{\left(l\right)}\right)$$

(2) $$X^{\left(l\right)}=\text{MaxPooling}\left({\hat{X}}^{\left(l\right)},s\right).$$

Here, *F^(l)^* is the number of feature maps at layer *l*, *T^(l)^* is the length of the sequence at layer *l*, *W^(l)^* ∈ ℝ^*F(l)* × *c* × *F*(*l*-1)^ and *b*^(*l*)^ ∈ ℝ^*F(l)*^ represent the convolutional filter parameters at layer *l, c* is the kernel size, *f* is the Rectified Linear Unit (ReLU), $X_{t-\frac{c}{2}-1:t+\frac{c}{2}}^{\left(l-1\right)}$ is a temporal section of the previous layer’s activation, ${\hat{X}}^{\left(l\right)}\in {\mathbb{R}}^{F^{\left(l\right)}\times T^{\left(l-1\right)}}$ is the temporal convolution output and *X^(l)^* ∈ ℝ^*F*(*l*) ×*T*(*l*)^ is the max pooling output with stride *s*, where $T^{\left(l\right)}=\frac{T^{\left(l-1\right)}}{s}$. When real-time evaluation is required, acausal convolutions (equation 1) are replaced with causal convolutions:

(3) $${\hat{X}}_{t}^{\left(l\right)}=f\left(W^{\left(l\right)}*X_{t-c-1:t}^{\left(l-1\right)}+b^{\left(l\right)}\right).$$

The decoder uses temporal convolutions and upsampling layers to gradually bring the data back to their original temporal resolution for frame-wise classification.

We also draw inspiration from temporal U-Net [41] and introduce shortcut connections between the two stages, where features from corresponding layers in the encoder and decoder are concatenated to allow the propagation of low level contextual information to the high level layers.

After each max pooling and feature concatenation layer, channel-wise feature normalization [21] as well as temporal dropout are employed for regularization.

### B Multimodal sensor fusion

#### 1) Baseline I - Concatenation TCN (C-TCN)

In order to fuse the two unimodal representations, the last layers from the kinematic and video streams are concatenated and projected through a fully-connected layer with softmax activation function to obtain action predictions.

#### 2) Baseline II - Ensemble TCN (E-TCN)

Alternatively, multimodal fusion can be obtained with plain ensemble of the two unimodal models, that is training the two streams with average unimodal loss and taking their average prediction probabilities as the final multimodal prediction.

#### 3) Multimodal Attention TCN (MA-TCN)

**Fig. 7:**
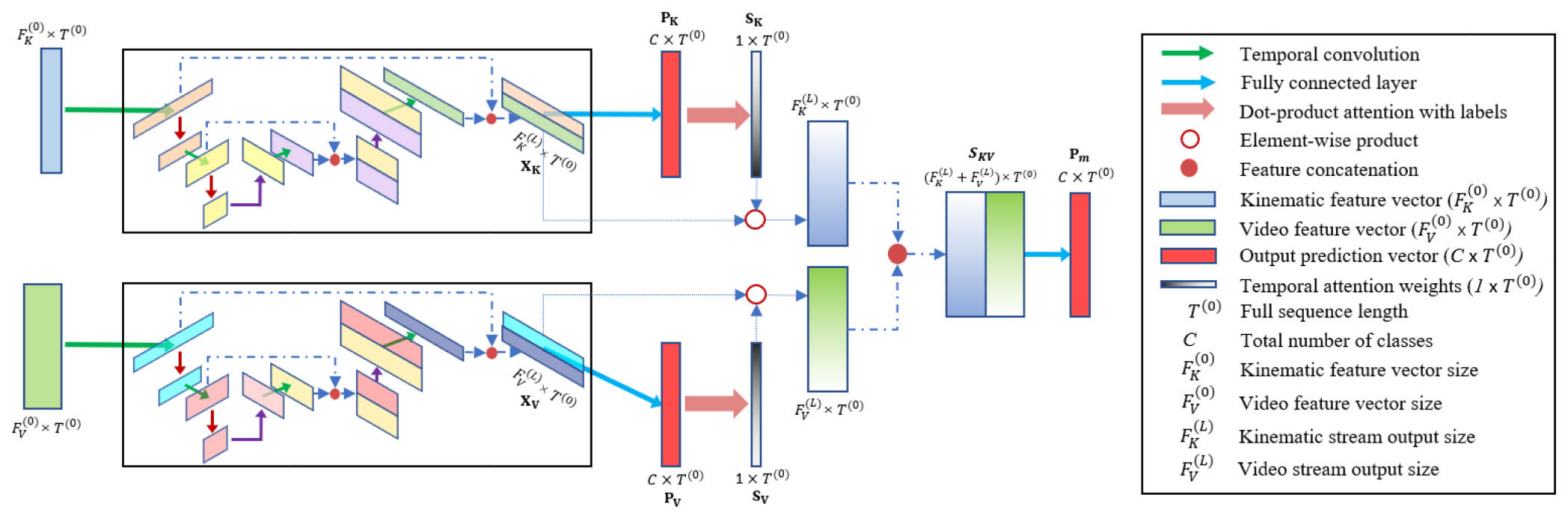
MA-TCN schematic. Starting from our two unimodal baselines, we derive frame-wise reliability weights for each stream using multimodal attention. During training, the output of each unimodal stream is compared to the corresponding one-hot-encoded label sequence through dot-product attention. The resulting scores are used as reliability weights to balance the relative contribution of different modalities. Action predictions are obtained by weighted concatenation of the final layers from the two streams followed by a fully-connected layer with softmax activation function.

Video and kinematic data carry complementary information which could be useful to understand each action’s internal dynamics. They are also subject to different noise sources that often manifest erratically. Recognition of surgical actions could then be improved by highlighting or penalizing the contribution of each modality dynamically in time. Building on E-TCN and C-TCN, we derive frame-wise reliability weights for each stream using multimodal attention. During training (Fig. 7), the last decoder activation *X_u_* ∈ ℝ^*F^(L)^* × *T*^(0)^^ of each unimodal stream *u* ∈ {*K*, *V*} is transformed into *P_u_* ∈ ℝ^*C*×*T*^(0)^^ to match the number of classes *C*:

(4) $$P_{u}=\text{Softmax}\left(W_{u}X_{u}+b_{u}\right),$$

where *W_u_* and *b_u_* are linear projection parameters. *P_u_* is then compared to the corresponding one-hot-encoded label sequence *Y* ∈ ℝ^*C*×*T*^(0)^^ through scaled dot-product attention [16], where *Y_t_* ∈ ℝ^*C*×1^ represents the frame-wise attention queries and *P_ut_* ∈ ℝ^*C*×1^ the frame-wise attention keys:

(5) $$S_{ut}\left(Y,P_{u}\right)=\frac{Y_{t}^{⊤}P_{ut}}{\sqrt{C}},0\leq t\leq T^{\left(0\right)}.$$

The resulting scores *S_u_* ∈ ℝ^1 ×*T*(0)^ measure the similarity between unimodal predictions and corresponding ground truth at each time-stamp and are used as reliability weights to balance the relative contribution of different modalities. After normalization [12]:

(6) $$S_{u}^{\prime }=\frac{e^{S_{u}}}{\sum_{u}e^{S_{u}}},$$

the generated weights $S_{ut}^{\prime }$ are thus multiplied with the outputs of the corresponding stream *X_ut_*, representing the frame-wise attention values. Multimodal action predictions *P_m_* ∈ ℝ^*C*×*T*^(0)^^ are then obtained by concatenation of the weighted outputs $S_{ut}^{\prime }X_{ut}$ from the two streams followed by a fully-connected layer with *W_m_* and *b_m_* parameters and softmax activation function:

(7) $$S_{KVt}=\left[S_{Kt}^{\prime }X_{Kt}∥S_{Vt}^{\prime }X_{Vt}\right],0\leq t\leq T^{\left(0\right)}$$

(8) $$P_{m}=\text{Softmax}\left(W_{m}S_{KV}+b_{m}\right).$$

It is worth noting that computation of attention weights is performed for each frame individually, thus allowing online processing of the input sequences when causal temporal convolutions are employed in the unimodal streams.

### C Input embeddings

Both unimodal streams operate on high-level feature sequences derived from the original data. As for JIGSAWS, we followed the majority of related work to aid comparability and used high-level visual features $\left(F_{V}^{\left(0\right)}=128\right)$ extracted from the raw video frames with a spatial CNN [19] along with smoothed and normalized selection of kinematic signals (positions, linear velocities and gripper angles) recorded from the two PSMs $\left(F_{K}^{\left(0\right)}=28\right)$.

The RARP-45 raw data were processed in a similar manner. Kinematic signals (positions, orientations and joint angles) recorded from two out of three PSMs $\left(F_{K}^{\left(0\right)}=69\right)$ were smoothed and normalized. High-level spatial features $\left(F_{V}^{\left(0\right)}=512\right)$ were extracted from the original video frames with ResNet18 [42] fine-tuned on the task of gesture recognition. Fine-tuning was performed on the training data via cross-validation, in order not to observe any test sequence during feature extraction.

Both datasets were down-sampled to 5Hz to reduce data redundancy and computation load.

### D Training and inference

The goal of our network is to optimize predictions from both unimodal streams and simultaneously exploit the third multimodal branch to down-weight noisy features that could not be rectified with unimodal training due to sensor-specific noise and limitations. We therefore trained our network using a weighted combination of unimodal (*L_k_*, *L_v_*) and multimodal (*L_kv_*) cross-entropy losses:

(9) $$L=L_{kv}\left(P_{m}\right)+w_{1}*L_{k}\left(P_{K}\right)+w_{2}*L_{v}\left(P_{V}\right)$$

where *w*_1_ and *w*_2_ represent balancing weights for the unimodal losses. Loss values around the gesture boundaries were down-weighted to compensate for smooth transitions and annotation uncertainty (see Section V-B).

As for inference, it is not possible to use action labels in order to obtain attention scores. We thus monitored the recognition scores on the validation sets to decide when to stop attention-based training, and then fine-tuned the uni-modal branches using average unimodal loss without attention, conjecturing that attention-based pre-training could improve feature robustness and increase recognition accuracy. After fine-tuning, inference could be performed readily as in our baseline.

## V Experiments and Results

### A Evaluation protocol

#### 1) JIGSAWS Dataset

We used the standard Leave-One-User-Out (LOUO) cross-validation setup, consisting of eight validation folds featuring all trials performed by the same user. The LOUO setup penalizes overfitting to user-specific features and it is useful to evaluate model generalization to different surgeons. As no independent test set is available, the network performance was defined as the average accuracy over crossvalidation splits.

#### 2) RARP-45 Dataset

We first divided the dataset into two parts with balanced class proportions, one for training (about 80%) and one for testing (about 20%) (Fig. 4c). Given the limited dataset size, we further divided the training set into 4 sub-sets and performed cross-validation for parameter tuning, estimating the optimal number of training epochs. The entire training set was then used to re-train the network and evaluate its performance on the unobserved test set.

#### 3) Evaluation metrics

Results were analysed based on three most common evaluation metrics: accuracy, Edit score and F1@10 score. Accuracy is used to test frame-wise recognition performance, but it is not appropriate to assess temporal properties of the generated predictions, which might show similar accuracy but large qualitative differences. Edit and F1@10 scores are therefore used to evaluate network understanding of action ordering and task structure. While Edit score represents a distance between true and predicted label sequences, assessing action ordering without timing, F1@10 additionally examines the temporal overlap between predictions and ground truth segments of the same class. Detailed description of the evaluation metrics and their implementation details are reported in [43].

#### B Implementation details

For both datasets we used a 3-layer encoder-decoder video stream with output dimensions {64, 96, 96}{96, 64, 64}and a 2-layer kinematic stream with dimensions {64, 96}{96, 64}, both with max pooling stride *s* =2 and dropout rate *p* = 0.3. Temporal convolutions were performed at each time step (*t*) from *t* — 24 to *t* + 25 (kernel size *c* = 50) in acausal experiments, and from *t* — 24 to *t* (kernel size *c* = 25) in causal experiments. symmetric windows of temporal weights *W_trans_* = [1,0.9,0.5,0.5,0.9,1] were centered around each transition point and multiplied with the loss samples, while both *w*_1_ and *w*_2_ balancing weights were heuristically set to 0.25. optimization was performed with the Adam optimizer (*α* = 0.9, *β* = 0.98) and a learning rate of 0.0005.

The network was implemented in PyTorch 1.5.0 and trained on NVIDIA Tesla V100-DGXS GPU.

#### C Results on JIGSAWS

##### 1) Acausal experiments

We first performed acausal experiments to evaluate our network’s best possible performance. Results of the ablation study are reported in Table II. Following [15], we trained each configuration three times with different initial seeds and recorded average scores for each validation split. We then reported the mean and standard deviation of the performance metrics across all 8 validation splits.

Loss down-weighting around the transition points (W_trans_) led our model to higher segmental scores, so we used it to train all the models. While our multimodal baselines (E-TCN, C-TCN) already showed better results compared to both unimodal streams (V-TCN and K-TCN), further improvement was obtained with multimodal attention (MA-TCN). In order to support our analysis statistically, we first used Kolmogorov-Smirnov test to verify if the cross-validation score vectors were normally distributed; as the scores were not normally distributed, we compared MA-TCN to the two multimodal baselines using two-sided Wilcoxon signed rank test with *α* = 0.05 cutoff for significance. Results demonstrated a significant difference in accuracy with C-TCN, and a significant difference in all the scores with E-TCN.

**Fig. 8:**
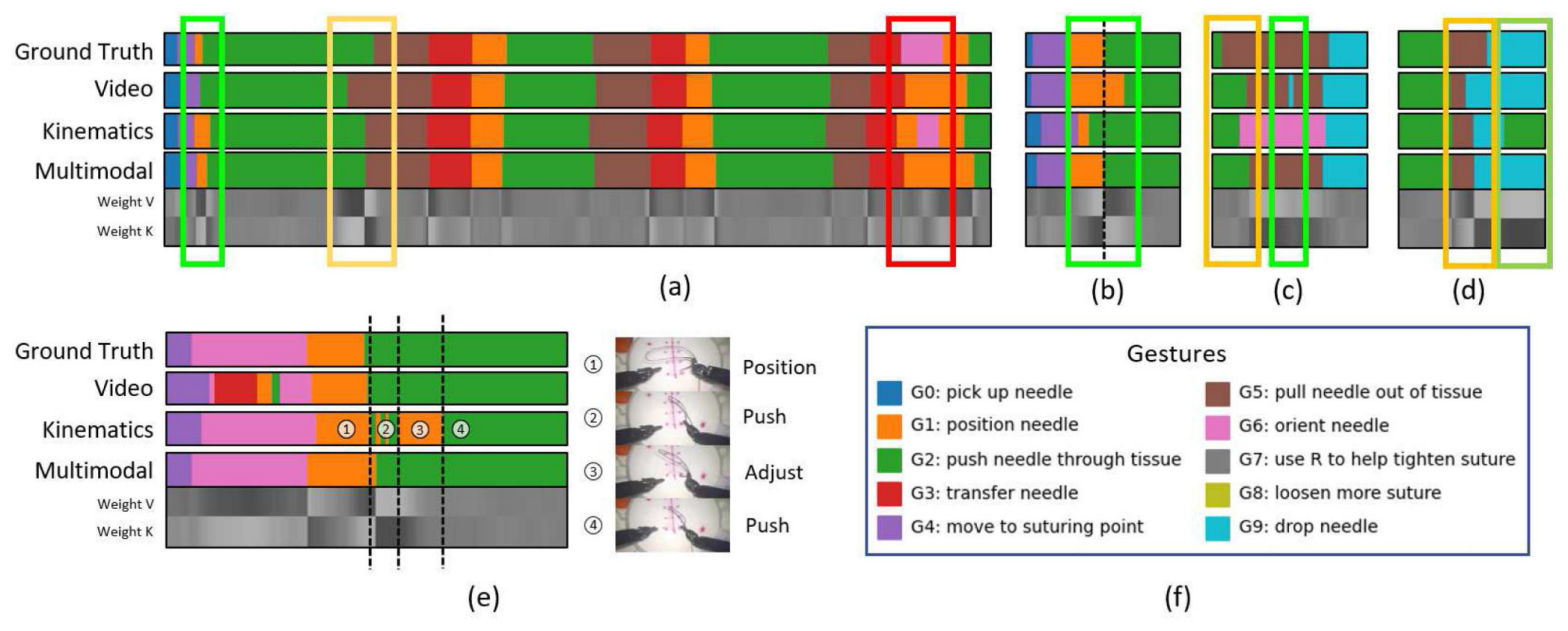
Examples of MA-TCN prediction outputs before fine-tuning and corresponding attention weights (gray scale representation, white = 0.65, black = 0.35), aligned with ground truth and unimodal predictions. Green boxes highlight instances where MA-TCN correctly enhances information from the most reliable modality at each time-stamp, recovering missed segments (a), improving boundary adherence (b), ignoring spurious segments (d) or rectifying simultaneous classification errors (c). In yellow we highlight when MA-TCN’s performance is limited by the accuracy of the strongest modality. The red box shows an example of failure mode, where higher weight is assigned to the weakest modality. (e) Physical interpretation of unimodal classification errors and corresponding attention weights. While gesture G6 (orient needle) is visually similar to G3 (transfer needle) and can benefit from higher kinematic attention to prevent misclassification, gestures G1 (position needle) and G2 (push needle through tissue) have similar kinematic properties when multiple adjustment motions are performed during G2. Using visual information to identify when the needle tip is inside the tissue leads to improved recognition.

Examples of MA-TCN prediction outputs before fine-tuning and corresponding attention weights, aligned with ground truth and unimodal predictions, are shown in Fig. 8 Green boxes highlight instances where MA-TCN correctly enhances information from the most reliable modality at each time-stamp, recovering missed segments (a), improving boundary adherence (b) or ignoring spurious segments (d). In few instances it is even able to rectify simultaneous classification errors (c). In other instances, however, MA-TCN’s performance is limited by the accuracy of the strongest modality (yellow box). An example of failure mode is highlighted in red, where higher weight is assigned to the weakest modality.

The interaction between video and kinematic predictions and corresponding attention weights sometimes finds physical interpretation, as illustrated in Fig. 8d. While gesture G6 (orient needle) is visually similar to G3 (transfer needle) and can benefit from higher kinematic attention to prevent misclassification, gestures G1 (position needle) and G2 (push needle through tissue) have similar kinematic properties when multiple adjustment motions are performed during G2. Placing the focus on visual cues to identify when the needle tip is inside the tissue can be helpful to identify the boundary between the two gestures.

**Fig. 9:**
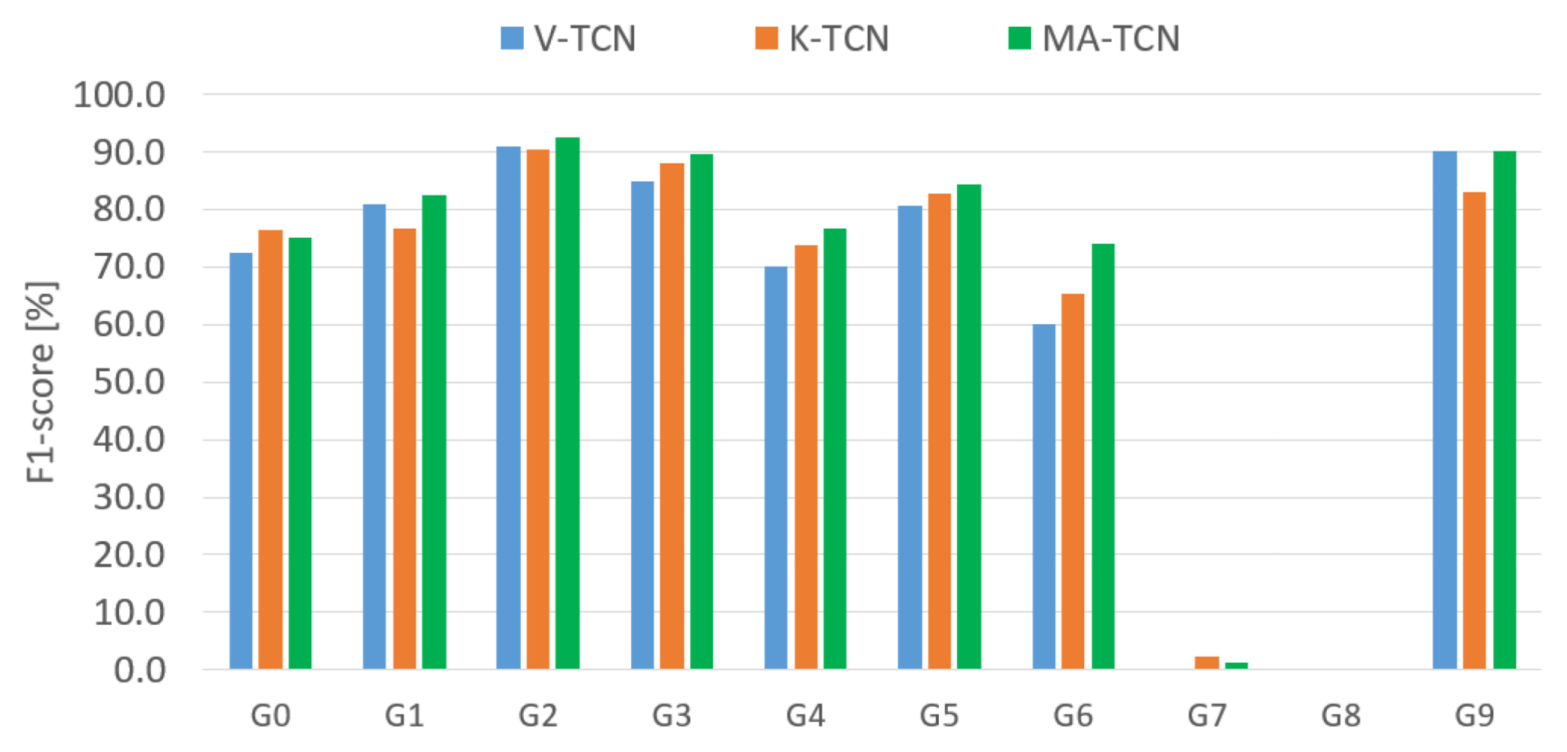
Per-class F1-scores on the JIGSAWS dataset.

Analysis of per-class F1-scores (Fig. 9) further confirms the observed behaviour, as MA-TCN scores are generally better than both modalities or at least better than the weakest modality. Gestures G7 and G8 are under-represented and thus remain very difficult to recognize. It is finally worth noting that MA-TCN generally shows better understanding of action ordering and task structure, which is only marginally reflected in the frame-wise evaluation scores (global and perclass accuracy).

##### 2) Comparison with related work

We compared acausal MA-TCN with related work aimed at optimizing multimodal fusion of video and kinematic data (Table III) for surgical gesture recognition. For fair comparison, we re-tested our network against the original label sequences (without rectification of the annotation errors) and reported new scores. Despite reporting lower average accuracy, MA-TCN shows superior performance in terms of segmental scores (Edit), indicating better ability to retrieve missing segments or delete spurious predictions from different data modalities. Moreover, [14] and [15] use more complex baselines with three unimodal streams (two for the kinematics and one for the video) embedding both temporal convolutions and recurrent cells. Similar strategies could help to improve MA-TCN’s overall accuracy.

##### 3) Causal experiments

We repeated our ablation study in a causal scenario, where temporal convolutions are causal (equation 3) and predictions at each time stamp are only function of past and current data samples, thus allowing realtime application. MA-TCN still outperforms all unimodal and multimodal baselines (Table IV), with statistically significant difference in accuracy with respect to both C-TCN and E-TCN. Segmental scores show the largest drop compared to acausal recognition, as knowledge of near-future dynamics in acausal frameworks helps to regularize the predictions’ structure. While helping to improve the recognition accuracy, the use of multi-modal attention could not significantly compensate for the structural uncertainty in causal scenarios.

#### D Results on RARP-45

We performed acausal experiments on the RARP-45 dataset to evaluate our network’s performance in a more challenging real-case scenario. As reported in Table V, MA-TCN outperforms all the baselines in accuracy and F1@10 score on the test set.

**Fig. 10:**
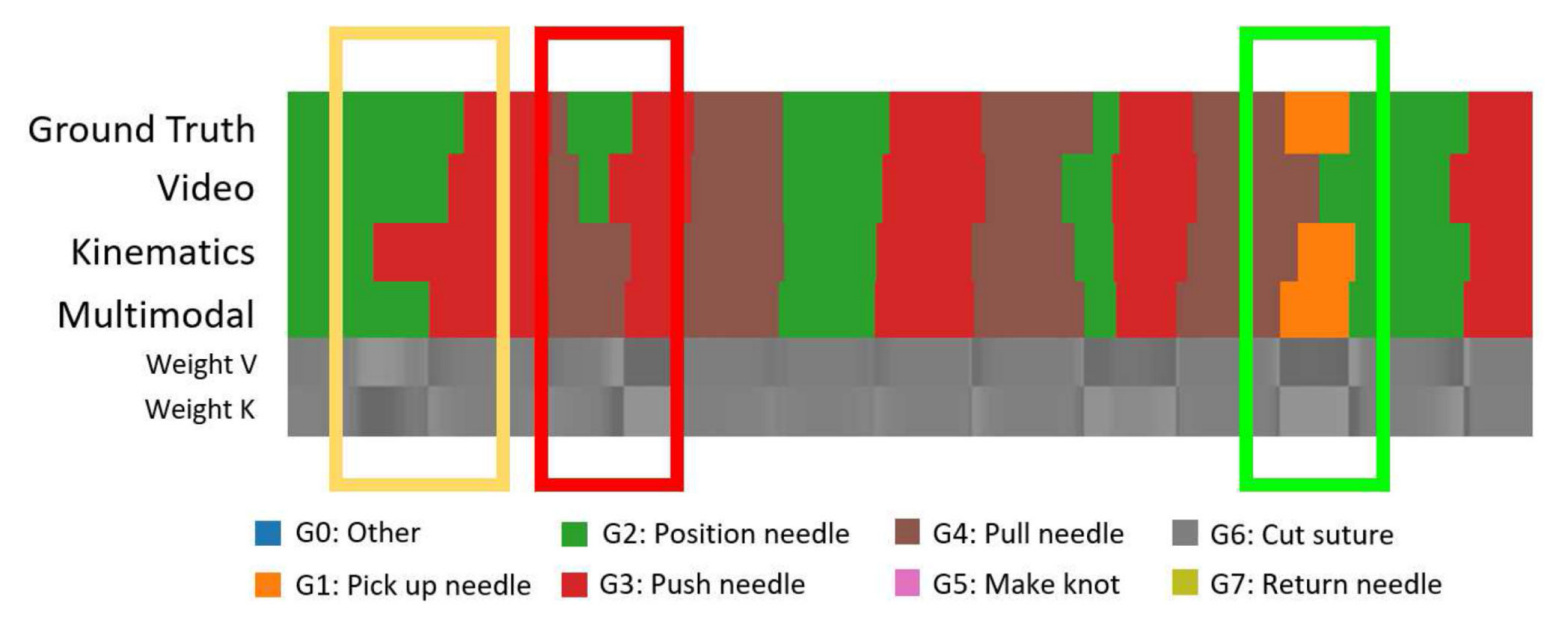
Examples of MA-TCN prediction outputs before fine-tuning and corresponding attention weights (gray scale representation, white = 0.65, black = 0.35), aligned with ground truth and unimodal predictions on the RARP-45 test set. The green box shows an example where MA-TCN correctly enhances information from the most reliable modality and outperforms both unimodal predictions, while the yellow box shows an example where MA-TCN’s performance is only better than the weakest modality. The red box shows an example of failure mode.

**Fig. 11:**
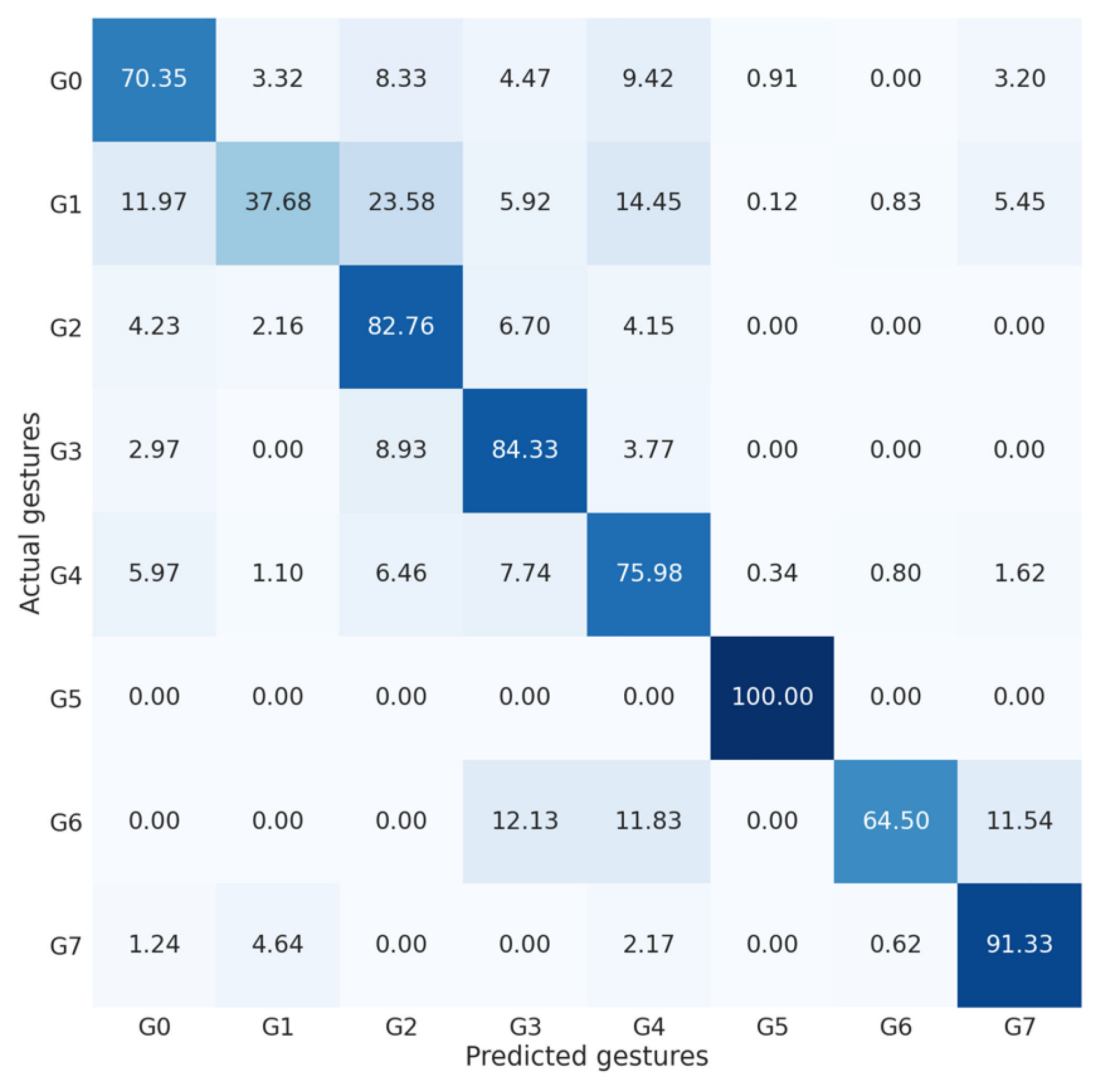
Normalized confusion matrix on the RARP-45 test set.

Fig. 10 shows examples of MA-TCN prediction outputs before fine-tuning and corresponding attention weights, highlighting successful and unsuccessful modality integration. As represented in the normalized confusion matrix (Fig. 11), a relevant percentage of the prediction errors falls at the gesture boundaries, where manual annotations are generally less accurate. Less frequent gestures such as G1 and G6 are sometimes missed and integrated into their temporally proximal segments (G0, G2 or G4 for G1, G4 or G7 for G6). Gesture G5 is recognized very well, but it only appears in a single test sequence. More data are needed to mitigate such strong class imbalance and perform robust training and evaluation.

**Fig. 12:**
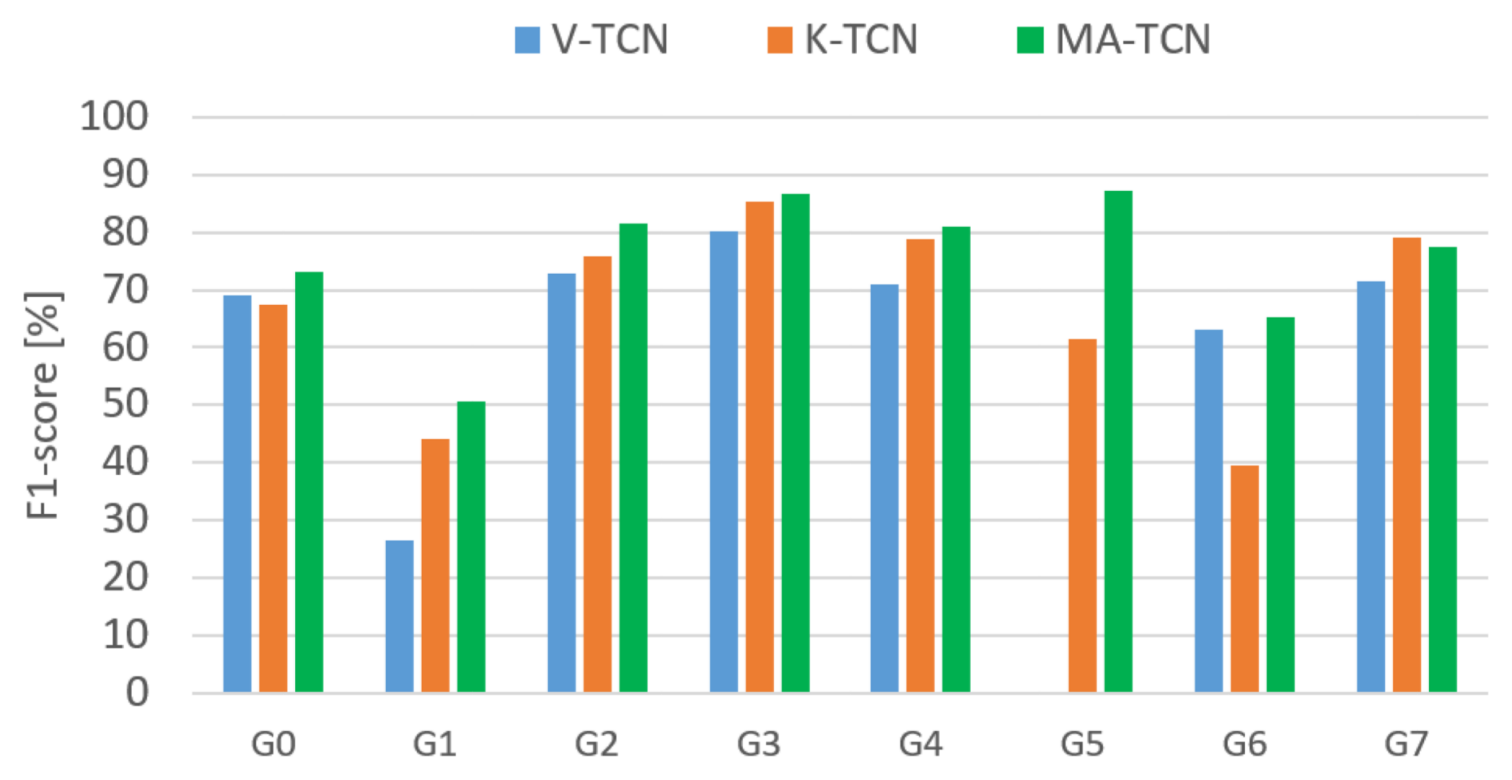
Per-class F1-scores on the RARP-45 test set.

Multimodal per-class F1-scores (Fig. 12) outperform both modalities for almost all classes. The visual stream is in most cases less robust than the kinematic stream, but the integration of low-level feature extraction through end-to-end training could partially compensate for the gap in performance.

## VI Conclusion

In this paper, we investigated using multimodal attention mechanisms to aid training of a two-stream network for surgical gesture recognition and achieve effective sensor fusion. The contribution of robot kinematics and visual information is balanced dynamically in time based on individual predictive power, resulting in combined prediction sequences with higher accuracy and better temporal structure. Visualization of attention weights also gives physically interpretable insights on network understanding of strengths and weaknesses of each sensor and modality.

Unlike related work, we tested our system on suturing demonstrations from real surgical interventions, where the complexity of the surgical environment and larger number of noise sources and variability factors makes fine-grained analysis and multimodal fusion particularly challenging. This is especially valuable for surgical-data-science translational research and for understanding the utilization of gesture recognition systems on real surgical data.

Method improvement is however needed to compensate for strong class imbalance in the available datasets, affecting the detection of infrequent classes, and to improve overall recognition accuracy on real surgical data, which is currently far from deployability levels. More real surgical data will also be collected to mitigate the problem, including different surgical phases (e.g. urethrovesical anastomosis) to introduce contextual data variability.

Surgical action characterization and definition of unambiguous gesture dictionary is another open problem itself [44], especially for bimanual operations. Available datasets like JIGSAWS have treated surgical demonstrations as singleaction sequences peformed by either robotic arm, ignoring the motion of the other arm. While this allows for faster labelling and simpler recognition models, it creates uncertainty when different gestures are performed simultaneously. In future work we aim to resolve such ambiguity with multi-label analysis and parallel recognition of right and left gestures, which is burdensome but more accurate and can account for action compositions [6]. Robust understanding of bimanual workflow and cooperation can help assessing surgical skill and is fundamental to achieve automation of complex action sequences in real surgical scenarios. Annotation variability studies based on multiple observers will also be performed to assess annotation accuracy and harmonize label sequences.

Beyond its limitations, the method also offers various possibilities of expansion and improvement. Similar attention modules could be placed on top of different layers to achieve adaptive sensor fusion at multiple abstraction levels. New data streams could also be added to enrich action representation and explore more complex multimodal interactions, using either new sensors (e.g. system event information) or new data streams automatically derived from available data (e.g. optical flow, semantic visual features, separated right and left instrument kinematics).

Future work will be finally aimed at integrating low-level visual feature extraction through end-to-end training, which is generally difficult on small datasets like JIGSAWS, as well as exploring similar attention mechanisms in the spatial [45] and temporal [26] domains.

## Supplementary Material

supp1-3147640

## Figures and Tables

**Table I:** RARP-45 dataset gesture list

ID	Gesture description	Count
G0	Background class	189
G1	Picking-up the needle	213
G2	Positioning the needle tip	535
G3	Pushing the needle through the tissue	500
G4	Pulling the needle out of the tissue	486
G5	Tying a knot	25
G6	Cutting the suture	35
G7	Returning/dropping the needle	71

**Table II:** Ablation study on JIGSAWS dataset - Acausal.

	Accuracy	Edit	F1@10
**K-TCN**	83.8 (5.4)	86.3 (5.7)	90.4 (4.0)
**V-TCN**	83.7 (6.1)	87.4 (6.1)	91.9 (4.0)
**C-TCN**	86.1 (5.3)	90.5 (5.4)	93.9 (3.6)
**E-TCN**	86.2 (5.2)	89.4 (5.0)	93.1 (3.5)
**MA-TCN w/o Wtrans**	86.6 (5.4)	90.1 (6.2)	93.6 (4.1)
**MA-TCN**	**86.8** (5.3)	**91.4** (6.3)	**94.3** (4.2)

**Table III:** Comparison with related work (original labels).

	Accuracy	Edit	F1@10
**Fusion-KV [14] (2020)**	86.3 (-)	87.2 (-)	-
**MRG-Net [15] (2020)**	**87.9 (4.2)**	89.3 (5.2)	-
**MA-TCN**	85.8 (5.1)	**90.3 (6.4)**	**93.6 (4.3)**

**Table IV:** Ablation study on JIGSAWS dataset - Causal.

	Accuracy	Edit	F1@10
**V-TCN**	81.5 (6.4)	73.0 (8.0)	82.0 (6.1)
**K-TCN**	76.7 (7.2)	74.4 (6.6)	81.5 (5.1)
**C-TCN**	82.3 (6.3)	**82.1 (5.5)**	**88.0 (4.4)**
**E-TCN**	82.6 (7.0)	81.4 (7.2)	87.3 (5.5)
**MA-TCN**	**83.4 (5.8)**	81.6 (7.6)	87.7 (5.3)

**Table V:** Results on real surgical data.

	Accuracy	Edit	F1@10
**V-TCN**	72.6	79.7	81.9
**K-TCN**	77.0	77.8	82.1
**E-TCN**	78.1	**80.3**	82.9
**C-TCN**	79.3	78.2	81.7
**MA-TCN**	**80.9**	79.6	**83.7**

## Footnotes

1 The data for this study were collected with participants’ consent as part of clinical service evaluation and were shared anonymously with researchers within the team in order to provide independent assessment of surgical performance.

## References

[1] Moorthy K (2004). Dexterity enhancement with robotic surgery. Surgical Endoscopy and Other Interventional Techniques.

[2] Chadebecq F, Vasconcelos F, Mazomenos E, Stoyanov D (2020). Computer vision in the surgical operating room. Visceral Medicine.

[3] Gao Y (2014). JHU-ISI Gesture and Skill Assessment Working Set (JIGSAWS): A Surgical Activity Dataset for Human Motion Modeling.

[4] Maier-Hein L (2020). Surgical data science-from concepts to clinical translation. arXiv preprint.

[5] Vedula SS (2016). Analysis of the structure of surgical activity for a suturing and knot-tying task. PloS one.

[6] Chen J (2018). Use of automated performance metrics to measure surgeon performance during robotic vesicourethral anastomosis and methodical development of a training tutorial. The Journal of urology.

[7] Nagy TD, Haidegger T (2019). A dvrk-based framework for surgical subtask automation. Acta Polytechnica Hungarica.

[8] Yasar MS, Alemzadeh H (2020). Real-time context-aware detection of unsafe events in robot-assisted surgery.

[9] De Rossi G (2019). Cognitive robotic architecture for semi-autonomous execution of manipulation tasks in a surgical environment.

[10] Schlomm T (2011). Full functional-length urethral sphincter preservation during radical prostatectomy. European urology.

[11] Ahmidi N (2017). A Dataset and Benchmarks for Segmentation and Recognition of Gestures in Robotic Surgery. IEEE Trans on Biomedical Engineering.

[12] Hori C (2017). Attention-based multimodal fusion for video description.

[13] Yang X, Ramesh P, Chitta R, Madhvanath S, Bernal EA, Luo J (2017). Deep multimodal representation learning from temporal data.

[14] Qin Y (2020). Temporal segmentation of surgical sub-tasks through deep learning with multiple data sources.

[15] Long Y-H (2020). Relational graph learning on visual and kinematics embeddings for accurate gesture recognition in robotic surgery. arXiv preprint.

[16] Vaswani A (2017). Attention is all you need. arXiv preprint.

[17] Varadarajan B, Reiley C, Lin H, Khudanpur S, Hager G (2009). Data-derived models for segmentation with application to surgical assessment and training.

[18] Lea C, Vidal R, Hager GD (2016). Learning convolutional action primitives for fine-grained action recognition.

[19] Lea C, Reiter A, Vidal R, Hager GD (2016). Segmental spatiotemporal cnns for fine-grained action segmentation.

[20] Funke I, Bodenstedt S, Oehme F, von Bechtolsheim F, Weitz J, Speidel S (2019). Using 3d convolutional neural networks to learn spatiotemporal features for automatic surgical gesture recognition in video.

[21] Lea C, Flynn MD, Vidal R, Reiter A, Hager GD (2017). Temporal convolutional networks for action segmentation and detection.

[22] Lea C, Vidal R, Reiter A, Hager GD (2016). Temporal convolutional networks: A unified approach to action segmentation.

[23] Lei P, Todorovic S (2018). Temporal deformable residual networks for action segmentation in videos.

[24] Wang J, Du Z, Li A, Wang Y (2019). Atrous temporal convolutional network for video action segmentation.

[25] Wang T, Wang Y, Li M (2020). Towards accurate and interpretable surgical skill assessment: A video-based method incorporating recognized surgical gestures and skill levels.

[26] Zhang J (2020). Symmetric dilated convolution for surgical gesture recognition.

[27] DiPietro R (2019). Segmenting and classifying activities in robot-assisted surgery with recurrent neural networks. Int journal of computer assisted radiology and surgery.

[28] Gurcan I, Van Nguyen H (2019). Surgical activities recognition using multi-scale recurrent networks.

[29] Ding L, Xu C (2017). Tricornet: A hybrid temporal convolutional and recurrent network for video action segmentation. arXiv preprint.

[30] Zappella L, Haro BB, Hager G, Vidal R (2013). Surgical gesture classification from video and kinematic data. Medical image analysis.

[31] Sarikaya D, Guru KA, Corso JJ (2018). Joint surgical gesture and task classification with multi-task and multimodal learning. arXiv preprint.

[32] Murali A (2016). Tsc-dl: Unsupervised trajectory segmentation of multimodal surgical demonstrations with deep learning.

[33] Zhao H, Xie J, Shao Z, Qu Y, Guan Y, Tan J (2018). A fast unsupervised approach for multi-modality surgical trajectory segmentation. IEEE Access.

[34] Lea C, Hager GD, Vidal R (2015). An improved model for segmentation and recognition of fine-grained activities with application to surgical training tasks.

[35] Qin Y, Allan M, Yue Y, Burdick JW, Azizian M (2021). Learning invariant representation of tasks for robust surgical state estimation. arXiv preprint.

[36] Long X (2018). Multimodal keyless attention fusion for video classification.

[37] Xu J, Yao T, Zhang Y, Mei T (2017). Learning multimodal attention lstm networks for video captioning.

[38] Guthart GS, Salisbury JK (2000). The intuitive/sup tm/telesurgery system: overview and application.

[39] van Amsterdam B, Clarkson MJ, Stoyanov D (2020). Multi-task recurrent neural network for surgical gesture recognition and progress prediction.

[40] Ficarra V, Cavalleri S, Novara G, Aragona M, Artibani W (2007). Evidence from robot-assisted laparoscopic radical prostatectomy: a systematic review. European urology.

[41] Wang F, Song Y, Zhang J, Han J, Huang D (2019). Temporal unet: Sample level human action recognition using wifi. arXiv preprint.

[42] He K, Zhang X, Ren S, Sun J (2016). Deep residual learning for image recognition.

[43] van Amsterdam B, Clarkson M, Stoyanov D (2021). Gesture recognition in robotic surgery: a review.

[44] Meireles OR (2021). Sages consensus recommendations on an annotation framework for surgical video. Surgical endoscopy.

[45] Wang F (2017). Residual attention network for image classification.

